# Editorial Comment: The impact of perioperative complications on favorable outcomes after artificial urinary sphincter implantation for post-prostatectomy incontinence

**DOI:** 10.1590/S1677-5538.IBJU.2019.0526.1

**Published:** 2020-05-20

**Authors:** André Cavalcanti, Alex Schul

**Affiliations:** 1 Universidade Federal do Estado do Rio de Janeiro Rio de JaneiroRJ Brasil Universidade Federal do Estado do Rio de Janeiro - UNIRIO, Rio de Janeiro, RJ

## COMMENT

The Artificial Urinary Sphincter (AUS) is considered the gold standard treatment of non-neurogenic male urinary incontinence in several Guidelines ( 1 - 3 ). Despite the high rates of initial continence, a significant number of patients will need some type of revision, generally due to infection, urethral erosion, return of incontinence or mechanical problems ( 4 ). Failure rates and the need for revision are generally associated with patient characteristics and history of previous treatment - for the cancer or for urethral strictures. Several studies compared the long-term results of AUS implantation with age, radiation therapy, urethroplasty, AUS reimplantation, hypogonadism, use of corticosteroids, smoking and other potential risk factors ( 5 - 8 ). Clearly, the preoperative characterization of the patient and his clinical history are fundamental for the establishment of results and complications expectations, which must be properly discussed with the patient to achieve the best satisfaction rates.

On the other hand, it is also important to observe the impact of perioperative complications on the late results of the implants. Among these perioperative complications we can mention: surgical infection, urinary infection (UTI), bleeding with the hematoma formation, urinary retention and unrecognized intra-operative urethral lesions. The clinical practice and the current literature demonstrate that these complications are directly related to rates of early explantation ( 9 ), but there is a lack of information about the long-term impact. In this study, the authors analyze the impact of perioperative complications in a group of 105 men who underwent an AUS implantation, in high volume centers, with an average follow-up of 38 months, focusing on the rates of explanation, continence and quality of life ( 10 ). The authors observed that the perioperative UTI was an independent risk factor for device explantation. When analyzing long-term continence rates, there was no relationship with any type of perioperative complication. The patients’ quality of life was affected only by postoperative pain and obviously by the final result of continence.

The prevention of perioperative complications is essential to decrease the rates of explantation, as previously demonstrated, including a study by this group ( 11 ). This prevention involves the proper preoperative patient evaluation of, identification of risk factors and an appropriate surgical technique. Despite the importance of the UTI, as an independent factor in the rate of late explantation, we still do not have a standardization in the use of antibiotic prophylaxis, as demonstrated in the methodology of this multicenter study, where about 37% of patients used prophylaxis with a single dose against about 63% using antibiotics also in the postoperative period.

Despite the methodological limitations, also identified by the authors, this study highlights the importance of UTI as an isolated risk factor for long-term sphincter explantation, demonstrating the need for robust, prospective, multicenter studies, with a sufficient number of patients, to cover the gap of information regarding antibiotic prophylaxis in AUS implants - regimens, timing and use of antibiotic coating devices

waiting line is full and it is better that they say, “with this doctor, it didn’t hurt at all”.
